# Predictors of Lumpectomy Size after Breast-Conserving Surgery in Patients with Breast Cancer: A Retrospective Cohort Study

**DOI:** 10.1097/PRS.0000000000011085

**Published:** 2023-09-26

**Authors:** Seher Makineli, Rogier Strijbis, Jonathan Tsehaie, Pascal P. A. Schellekens, Maaike R. Moman, Liesbeth M. Veenendaal, Patrick I. Ferdinandus, Arjen J. Witkamp, Milan C. Richir, Wies Maarse

**Affiliations:** Utrecht and Bilthoven, the Netherlands; From the Departments of 1 General Surgery; 2 Plastic, Reconstructive, and Hand Surgery, University Medical Centre Utrecht; Departments of 3 Radiology; 4 General Surgery; 5 Plastic, Reconstructive, and Hand Surgery, Alexander Monro Hospital.

## Abstract

**Background:**

Oncoplastic reconstructive surgery as an extension of breast-conserving surgery leads to better aesthetic results, an increase in tumor-free margins, and a reduction in reexcision rates. Oncologic resection is often more extensive than expected, sometimes resulting in the plastic surgeon deviating from the predetermined plan. For optimal planning of the reconstruction, it is mandatory to estimate volume defects after lumpectomy as accurately as possible. The authors aimed to identify preoperative predictors of lumpectomy resection size.

**Methods:**

All consecutive patients diagnosed with invasive breast carcinoma or carcinoma in situ and treated primarily with breast-conserving surgery between 2018 and 2020 at the University Medical Center Utrecht or Alexander Monro Hospital were included. Patient and tumor characteristics were measured. Data were analyzed in a multiple linear regression analysis.

**Results:**

A total of 410 patients (423 cases) were included, with a median age of 58 years (range, 32 to 84 years) and a mean body mass index (BMI) of 25.0 (SD 9.3). The mean maximum radiologic tumor diameter was 18.0 mm (SD 13.2), and the mean maximum lumpectomy diameter was 58.8 mm (SD 19.2). Multiple linear regression analysis found an explained variance of R^2^ = 0.60 (*P* < 0.00), corrected for operating surgeon. Significant predictors for postoperative lumpectomy size were BMI, breast size, and maximum preoperative radiologic tumor diameter. A predictive tool for lumpectomy size was developed and a web-based application created to facilitate use of the tool in a clinical setting.

**Conclusions:**

Postoperative lumpectomy size can be predicted using BMI, breast size, and radiologic tumor size. This model could be beneficial for breast surgeons in planning reconstructions and preparing and informing their patients more accurately.

**CLINICAL QUESTION/LEVEL OF EVIDENCE:**

Risk, III.

Every year, around 15,000 women are diagnosed with invasive breast cancer and 2300 with carcinoma in situ in the Netherlands,^1^ making it the second most common cancer after skin cancer. In 2017, 60.1% of patients with breast cancer who underwent surgery had breast-conserving surgery (BCS).^2^ Several studies have shown that BCS combined with adjuvant radiotherapy is an adequate treatment for early-stage breast cancer, with comparable oncologic results to mastectomy, and resulting in better quality of life.^3–7^

The survival rate for breast cancer has improved substantially in recent decades.^8^ The 5- and 10-year relative survival increased from 76.8% and 55.9%, respectively, in 1989 through 1999 to 91.0% and 82.9% in 2010 through 2016.^2^ Because of this improvement, the focus of treatment has expanded from survival to include quality of life. In recent years, oncoplastic surgery (OPS)—BCS in combination with reconstruction—has improved both oncologic and reconstructive outcomes and has expanded indications for breast conservation.^9^ Research shows it is a safe alternative to BCS, both for small and large tumors.^10,11^ OPS has several advantages compared with BCS only and mastectomy. A more extensive resection can be performed without aesthetic limitations, the incidence of tumor-free margins has increased, and the need for reexcision has decreased. In addition, a decrease in conversion to a mastectomy, in which the complete breast tissue is fully removed, has occurred.^12,13^

For plastic surgeons, it is more relevant to estimate the actual lumpectomy size than the tumor size; hence, for reconstruction purposes, the estimated volume defect is most important. In general, oncologic surgeons try to maintain 1 cm of macroscopic free margin around the tumor during BCS.^14^ Nonetheless, research shows that the oncologic resection is often more extensive than expected, especially for smaller tumors.^15^ As a result, the plastic surgeon sometimes must deviate from the predetermined plan. Reconstruction techniques or extra scarring may be necessary that have not been properly discussed with the patient preoperatively or for which additional examination is desirable (eg, vascular examination for a pedicled flap). A better estimate of the size of the lumpectomy could help the plastic surgeon make a more reliable reconstructive plan.

No previous research has been conducted comparing preoperative tumor size with the actual size of the excised lumpectomy. This study aimed to identify which factors in addition to radiologic tumor size influence lumpectomy size, and provide a model to calculate the lumpectomy size preoperatively.

## PATIENTS AND METHODS

### Study Design

This retrospective cohort study was performed at University Medical Center Utrecht (UMCU) and Alexander Monro Hospital (AMH), a hospital specialized in breast cancer care in the Netherlands, and approved by the local institutional review board (no. 21/327). These hospitals work closely together in breast cancer care. Women clinically diagnosed with invasive breast carcinoma or carcinoma in situ were included if they had primary BCS between January 1, 2018, and December 31, 2020. Cases were filtered from the electronic health records (EHRs) by using the diagnosis treatment combination code.^16^ Patients undergoing surgery for benign lesions or malignant and benign lesions combined in the same breast were excluded. Other exclusion criteria were previous oncologic surgery to the same breast, previous breast augmentation, surgery resulting in 2 separate lumpectomies in 1 breast, surgery for Paget disease, or removing the tumor en bloc with the breast reduction tissue.

### Patient Characteristics

Patient, tumor, and surgery characteristics were obtained from the EHRs. Height, weight, body mass index (BMI), smoking status, and breast size were reported. Patients who had not smoked for at least 10 years were considered nonsmokers. Breast size was reported by the patient using European Union standards.^17^

### Preoperative Diagnostics

A stereotactic vacuum-assisted breast biopsy was performed to examine suspect lesions. They were reported as invasive ductal carcinoma, invasive ductolobular carcinoma, or invasive lobular carcinoma; ductal or lobular carcinoma in situ; or others (eg, papillary, tubular, or mucinous carcinoma).

All patients received radiologic breast examinations consisting of magnetic resonance imaging, ultrasound, mammography, or tomography. All studies were reviewed and reported by specialized radiologists. The maximum diameter of the area to be excised was recorded in the database for each radiologic modality if present. The modality with the largest measurement was used in the analysis. In case of neoadjuvant therapy, the measurements were determined before and after therapy.

Primary tumor, lymph node, and metastasis (TNM) classification was determined and recorded during multidisciplinary consultation in the presence of an oncologic surgeon, plastic surgeon, oncologist, radiologist, and pathologist, following the American Joint Committee on Cancer’s *Cancer Staging Manual, Eighth Edition*.^18^

### Surgery

BCS was performed by an oncologic surgeon, and in some cases, in combination with a plastic surgeon. Surgery characteristics were obtained from the surgery reports. Perioperative tumor localization was performed by palpation, wire localization, ultrasonography, or radio-guided occult lesion localization with iodine-125 seeds. The reconstructive technique was reported and divided into 4 categories: 1, primary closure (including local transposition); 2, volume rearrangement and skin correction (eg, round block, batwing, B-plasty); 3, volume/breast reduction; or 4, volume replacement. Additional surgery due to an incomplete resection was also reported.

### Pathology

All removed tissue was evaluated at UMCU by specialized pathologists. Reports were reviewed for the maximum diameter of the excised lump, maximum diameter of the tumor, radicality of the resection, and the presence of a shave. Resection was considered incomplete if the margins were more than focally positive.

### Statistical Analysis

Data were analyzed using R statistical computing, version 4.0.4 (R Core Team). Continuous data were described using mean with SD for normally distributed data, and the χ^2^ test was used for categorical values to assess differences between groups.

Analysis of variance and Pearson correlations were used to determine whether interaction effects between predictor variables existed. No strong interactions between variables were found (Pearson correlation higher than 0.15). Therefore, no interaction terms were added to the multiple regression analysis. The outcome was corrected for the surgeon and the hospital in the regression analysis. Missing data were handled using imputation with multiple imputation by chained equations.^19^ *P* values less than 0.05 were considered to be statistically significant.

## RESULTS

**Fig. 1.**
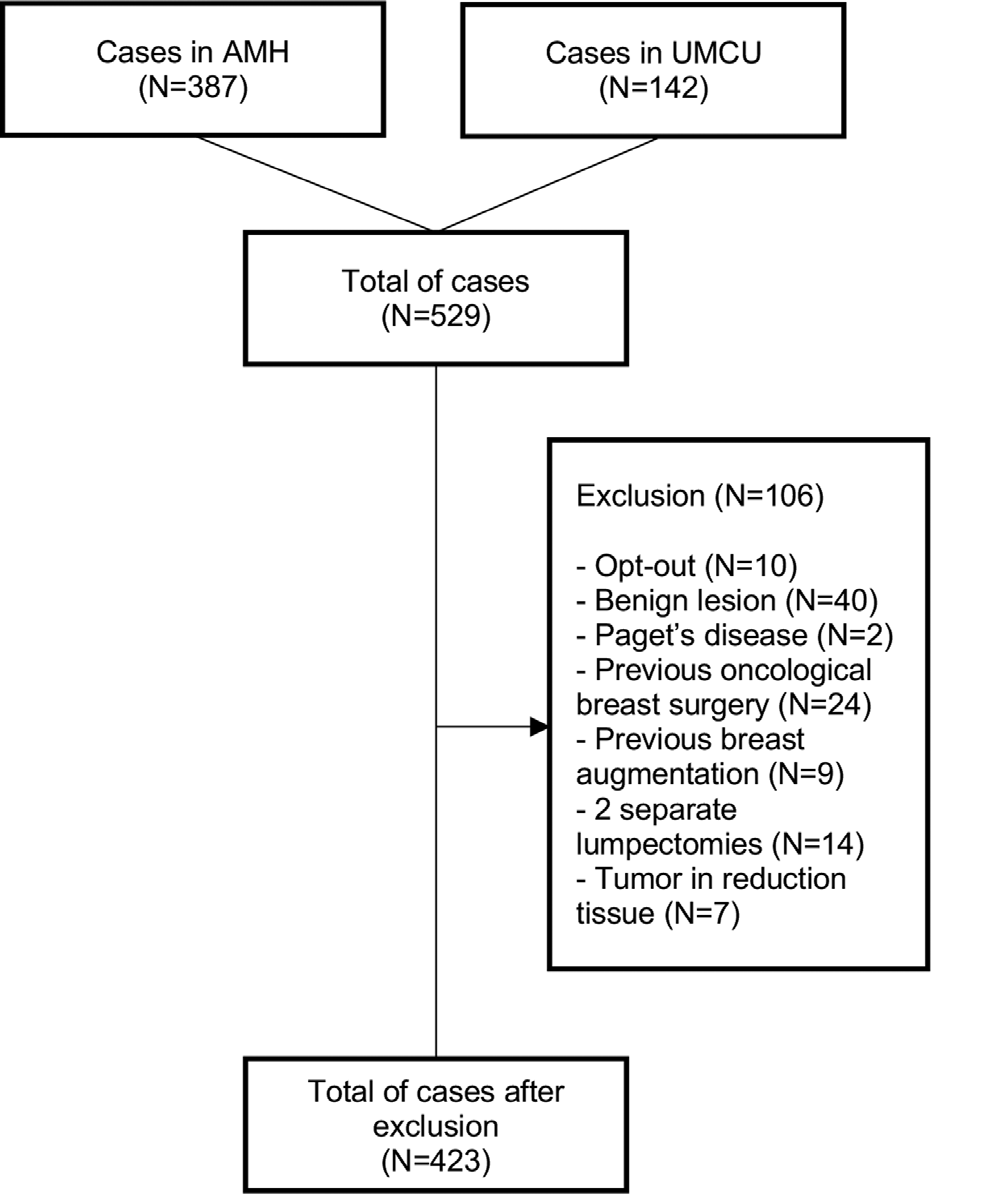
Flowchart of the study. *AMH*, Alexander Monro Hospital; *UMCU*, University Medical Center Utrecht.

A total of 529 cases (508 patients) were identified from the EHRs of AMH and UMCU. We excluded 106 cases (20.0%) for several reasons (Fig. 1). Ten patients (1.9%) objected to participating in pseudonymized research. Forty patients (7.6%) were excluded because of surgery for benign lesions or malignant and benign lesions combined, and 2 patients (0.4%) were excluded because of surgery for Paget disease. Twenty-four patients (4.5%) had already had previous oncologic breast surgery, and 9 patients (1.7%) had received breast augmentation. Fourteen patients (2.6%) had 2 separate lumpectomies in 1 breast, and in 7 cases (1.3%), the tumor was removed en bloc with the reduction tissue.

In total, 423 cases were eligible for analysis. Most cases were treated at AMH (76.1%) (Table 1). OPS was more common at AMH than UMCU (81.1% versus 13.9% of operations per hospital). The mean BMI was 25.0 (SD 9.3), the median age during surgery was 58 years (range, 32 to 84), and 13.5% of patients were smokers. Common breast sizes were C (25.1%), B (24.1%), and D (23.9%). Most tumors were in the upper outer quadrant (43.7%) and were diagnosed as invasive ductal carcinoma (53.7%) (Table 2). Most were categorized as cT1c (>10 mm, ≤20 mm) (35.9%) or cT2 (>20, ≤50 mm) (27.9%). The majority of cases did not have lymph-node metastases (cN0; 91.3%). The mean radiologic maximum diameter of the area to be excised was 18.0 mm (SD 13.2). A quarter of the cases (25.8%) received neoadjuvant therapy before surgery. Primary closure was the most common reconstruction technique (56.0%), followed by volume reduction (24.6%). The mean maximum lumpectomy diameter was 58.8 mm (SD 19.2), with no significant difference between the groups with and without a plastic surgeon involved (2-sample *t* test *P* = 0.559). Results showed no significant difference in incomplete resections if a plastic surgeon was involved (12.8% versus 6.9%; χ^2^ test *P* = 0.065).

**Table 1.** Patient Characteristics^a^

Characteristics	Values (*n* = 423)
Hospital, no. (%)	
Alexander Monro Hospital	322 (76.1)
Plastic surgeon involved	261 (81.1^a^)
University Medical Center Utrecht	101 (23.9)
Plastic surgeon involved	14 (13.9^a^)
Age, median (range), yrs	58 (32–84)
Height, mean ± SD, m	1.69 ± 6.6
Weight, mean ± SD, kg	71.4 ± 11.9
BMI, mean ± SD, kg/m^2^	25.0 ± 4.0
Smoking, no. (%)	57 (13.5)
Breast size, no. (%)	
A	21 (5.0)
B	102 (24.1)
C	106 (25.1)
D	101 (23.9)
E	50 (11.8)
F	20 (4.7)
G	8 (1.9)
H	3 (0.7)
I	2 (0.5)
Unknown	10 (2.4)

a Percentage of cases per hospital.

**Table 2.** Tumor and Surgical Characteristics

Characteristics	Total (*n* = 423)	Plastic Surgeon Involved		*P*
		No (*n* = 148)	Yes (*n* = 275)	
Tumor left side, no. (%)	215 (50.8)	82 (55.4)	133 (48.4)	0.167
Tumor localization, no. (%)				0.122
Upper inner quadrant	67 (15.8)	17 (11.5)	50 (18.2)	
Lower inner quadrant	44 (10.4)	17 (11.5)	27 (9.8)	
Upper outer quadrant	185 (43.7)	71 (48.0)	114 (41.5)	
Lower outer quadrant	72 (17.0)	22 (14.9)	50 (18.2)	
Central	13 (3.1)	3 (2.0)	10 (3.6)	
Overlapping	42 (9.9)	18 (12.2)	24 (8.7)	
Histologic finding, no. (%)				0.347
IDC	227 (53.7)	74 (50.0)	153 (55.6)	
IDLC	68 (16.1)	25 (16.9)	43 (15.6)	
ILC	34 (8.0)	10 (6.8)	24 (8.7)	
DCIS	68 (16.1)	28 (19.9)	40 (14.5)	
LCIS	5 (1.2)	4 (2.7)	1 (0.4)	
Other	21 (5.0)	7 (4.7)	14 (5.1)	
cT category, no. (%)^a^				0.001
In situ	74 (17.5)	32 (21.6)	42 (15.3)	
1a	9 (2.1)	6 (4.1)	3 (1.1)	
1b	63 (14.9)	32 (21.6)	31 (11.3)	
1c	152 (35.9)	49 (33.1)	103 (37.5)	
2	118 (27.9)	27 (18.2)	91 (33.1)	
3	6 (1.4)	2 (1.4)	4 (1.5)	
4	1 (0.2)	0 (0.0)	1 (0.4)	
cN category, no. (%)^a^				0.864
0	386 (91.3)	137 (92.6)	249 (90.5)	
1	29 (6.9)	9 (6.1)	20 (7.3)	
2	3 (0.7)	1 (0.7)	2 (0.7)	
3	5 (1.2)	1 (0.7)	4 (1.5)	
Neoadjuvant therapy, no. (%)	109 (25.8)	32 (21.6)	77 (28.0)	0.153
Maximum radiologic diameter of disease,^b^ mean ± SD, mm	18.0 ± 13.2	13.1 ± 8.9	20.7 ± 14.4	0.001
Localization technique, no. (%)^a^				0.001
Palpation	64 (15.1)	12 (8.1)	52 (18.9)	
Wire	258 (61.0)	51 (34.5)	207 (75.3)	
Ultrasound	4 (0.9)	0 (0.0)	4 (1.5)	
Iodine-125	98 (23.2)	85 (57.4)	13 (4.7)	
Category of reconstruction technique, no. (%)^c^				0.001
1	237 (56.0)	147 (99.3)	90 (32.7)	
2	68 (16.1)	1 (0.7)	67 (24.4)	
3	104 (24.6)	0 (0.0)	104 (37.8)	
4	14 (3.3)	0 (0.0)	14 (5.1)	
Shave, no. (%)	84 (19.9)	25 (16.9)	59 (21.5)	0.262
Maximum pathologic lumpectomy diameter, mean ± SD, mm	58.8 ± 19.2	59.6 ± 19.7	58.4 ± 18.8	0.559
Incomplete resection, no. (%)	38 (9.0)	19 (12.8)	19 (6.9)	0.065
Additional surgery, no. (%)	39 (9.2)	19 (12.8)	20 (7.3)	0.077

cN, clinical lymph node status; cT, clinical primary tumor size; DCIS, ductal carcinoma in situ; IDC, invasive ductal carcinoma; IDLC, invasive ductolobular carcinoma; ILC, invasive lobular carcinoma; LCIS, lobular carcinoma in situ; TNM, tumor, lymph node, and metastasis.

a TNM classification following the American Joint Committee on Cancer’s *Cancer Staging Manual, Eighth Edition* (Guiliano AG, Connolly JL, Edge SB, et al. Breast cancer: major changes in the American Joint Committee on Cancer Eighth Edition *Cancer Staging Manual*. *CA Cancer J Clin*. 2017;67:290–303).

b Maximum diameter of total area to be excised, including all foci of pathologically proven invasive tumor and carcinoma in situ and nonmass enhancement.

c 1: Primary closure; 2: volume rearrangement; 3: volume reduction; 4: volume replacement.

Table 3 shows the results of the multiple linear regression analysis for predictors for postoperative lumpectomy size. After correction for different surgeons, significant predictors for maximum postoperative lumpectomy size were BMI, age, breast size, maximum preoperative radiologic diameter, and involvement of plastic surgeon (see Table 3, model 1). (**See Figure, Supplemental Digital Content 1**, which demonstrates a visualization of model 1, the relationship between the observed and predicted lumpectomy size; x/y axis: postoperative lumpectomy size; observed [*blue*] versus predicted [*pink*], http://links.lww.com/PRS/G905. **See Figure, Supplemental Digital Content 2**, which demonstrates the goodness-of-fit analyses of the predicted versus observed lumpectomy size models, http://links.lww.com/PRS/G906.)

**Table 3.** Multiple Linear Regression Analysis, Predictors for Postoperative Lumpectomy Size

Predictors	Model 1 (R^2^ = 0.616)			Model 2 (R^2^ = 0.598)		
	β Coefficient	95% CI	*P*	β Coefficient	95% CI	*P*
Intercept	31.868	18.034, 45.703	<0.001	32.710	20.394, 45.026	<0.001
BMI	1.133	0.819, 1.446	<0.001	1.127	0.808, 1.446	<0.001
Age	0.140	0.013, 0.268	0.031	—	—	—
Breast size						
B	1.560	−3.499, 6.619	0.545	1.852	−3.316, 7.020	0.482
C	4.774	−0.278, 9.826	0.064	4.962	−0.187, 10.110	0.059
D	9.736	4.534, 14.938	<0.001	9.733	4.418, 15.047	<0.001
E	11.423	5.643, 17.204	<0.001	12.322	6.423, 18.221	<0.001
F	13.007	6.031, 19.983	<0.001	12.916	5.792, 20.039	<0.001
G	16.760	7.313, 26.206	<0.001	15.969	6.327, 25.610	0.001
H	34.983	20.234, 49.732	<0.001	34.710	19.632, 49.788	<0.001
I	27.611	10.118, 45.105	0.002	31.376	13.562, 49.190	<0.001
Maximum radiologic diameter^a^	0.540	0.446, 0.634	<0.001	0.597	0.504, 0.690	<0.001
No plastic surgeon	−7.095	−10.361, −3.829	<0.001	—	—	—

a Maximum diameter of total area to be excised on magnetic resonance imaging, ultrasound, or mammography, including all foci of pathologically proven invasive tumor and carcinoma in situ and nonmass enhancement.

After performing the analysis without including plastic surgery, age became an insignificant predictor and was removed from the analysis. BMI (β = 1.127; *P* < 0.001) and preoperative tumor size (β = 0.597; *P* < 0.001) were positively correlated with postoperative lumpectomy size. Breast size showed a variable significance in positive correlation, depending on the actual size (see Table 3). By using the significant β coefficients, we could make a prediction model in which the relationship between the observed and predicted lumpectomy size was determined by following formula:


$$\begin{array}{l} \begin{array}{lll} & lumpdiameter\left(mm\right)tobeexcised=32.710+BMI*1.127 & +breastsize\left(\#\right)+radiologictumorsize\left(mm\right)*0.597 \end{array} \end{array}$$

where the number sign (#) indicates breast size (A = 1, B = 2, C = 3, D = 4, E = 5, F = 6, G = 7, H = 8, I = 9). For example, for a patient with breast cancer with a BMI of 25, breast size of C, radiologic tumor size of 17 mm, the lump diameter (mm) to be excised would be 32.710 + (25 * 1.127) + 3 + (17 * 0.597) = 74 mm. The underlying statistical formula was also implemented in a web-based calculator, accessible at www.evidencio.com under the title “predicted lumpectomy size.”

## DISCUSSION

In this study, we found a significant correlation between postoperative lumpectomy size and BMI, breast size, and preoperative radiologic tumor size, corrected for the operating surgeon. Using multiple linear regression analysis, we constructed a formula that can be useful for surgeons during clinical practice. This study shows that postoperative lumpectomy size is often larger than just adding a 1-cm margin to the radiologic tumor size, a common practice to estimate lumpectomy size.

Although our study shows a significantly larger mean radiologic tumor diameter in the OPS group, no significant difference was found in mean postoperative lumpectomy size. It seems that tumors in the OPS group were removed with smaller margins. However, this is a biased result due to differences in the incidence of OPS between hospitals. Per hospital individually, both preoperative tumor size and postoperative lumpectomy size were larger in the OPS group.

There were more incomplete resections in the BCS group compared with the OPS group. However, this difference was not significant. Previous research stated that incomplete resections were less common in OPS.^9,12,13,20^

No previous research on predictors of postoperative lumpectomy size has been published. A partially comparable study investigated the relationship between tumor and lumpectomy volume.^15^ The outcome is consistent with our finding that the lumpectomy is often larger than the tumor plus 1 cm margin. However, the authors did not examine other predictors of lumpectomy size or volume. In addition, they assumed that the tumor and the lumpectomy had the shape of a perfect sphere and ellipsoid, respectively, to calculate volumes. This may not be a reliable estimation because research shows that only 19% of all breast tumors are spherical.^21^

Another retrospective cohort study did not find a significant correlation between intraoperative localization technique and lumpectomy size. They also used an estimated lumpectomy volume as an outcome.^22^ A multicenter randomized controlled trial found that ultrasound-guided surgery gave smaller excision volumes than palpation-guided surgery without an increase of incomplete resections.^23^ The authors measured the volume directly after excision in the operating theater by using the fluid displacement technique.

A strength of this study is the extensive database. Many patients were included, and there were few missing data in the variables used for statistical analysis, which could be completed with multiple imputation by chained equations. In addition, this is the first study on predictors of postoperative lumpectomy size.

Several limitations should be noted. Due to the retrospective design, no additional tests or measurements could be done to complete the database. It was not possible to analyze the influence of breast ptosis or underbust circumference. There were differences in radiologic modality and intraoperative tumor localization. Therefore, it was not possible to investigate the influence of these factors on the outcomes. Another limitation is the use of breast cup size as volume estimation. Although many patients and plastic surgeons use “bra cup,” there is no uniform sizing standard. It is an arbitrary scale with interobserver variability and differences between countries and bra brands.^24^ In the absence of more reliable measurements and the retrospective design of this study, we used this means to estimate breast volume. In future studies, more reliable techniques, such as volumetric computed tomography or magnetic resonance imaging, water displacement, or three-dimensional photography, could be used.

To validate the accuracy of the lumpectomy size tool in estimating lumpectomy size before surgery, we are in the preparation phase of a prospective validation study. Given the prospective nature of the study, physicians can precisely assess and record ptosis, underbust circumference, cup size and volume measurement, weight, and height to collect current and reliable data. Furthermore, it will be interesting to investigate the correlation among lumpectomy size, radiologic modality, and intraoperative localization methods.

## CONCLUSIONS

Our study showed that postoperative lumpectomy size can be predicted in a simple model using BMI, breast size, and radiologic tumor size. The model could help plastic surgeons make reconstruction plans and better inform their patients.

## DISCLOSURE

The authors have no financial disclosures to report.

## Supplementary Material


