# Supplementary figures and images for: Predictors of Lumpectomy Size after Breast-Conserving Surgery in Patients with Breast Cancer: A Retrospective Cohort Study
Source: Plast Reconstr Surg. 2023 Sep 26;154(3):503–10. doi: 10.1097/PRS.0000000000011085 (PMC11346708; doi:10.1097/PRS.0000000000011085)

Predicted ◇  
Observed ◇

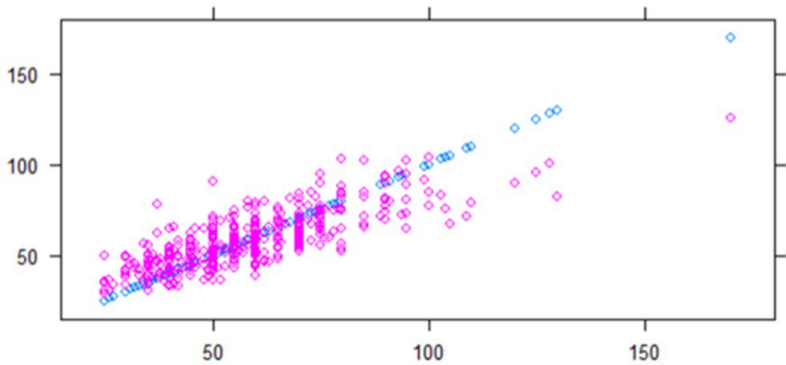

Supplement: Supplementary file 1 [file prs-154-0503-s001.pdf]
