# Supplementary material for: Predictors of Lumpectomy Size after Breast-Conserving Surgery in Patients with Breast Cancer: A Retrospective Cohort Study
Source: Plast Reconstr Surg. 2023 Sep 26;154(3):503–10. doi: 10.1097/PRS.0000000000011085 (PMC11346708; doi:10.1097/PRS.0000000000011085)
Supplement: Supplementary file 2 [file prs-154-0503-s002.pdf]

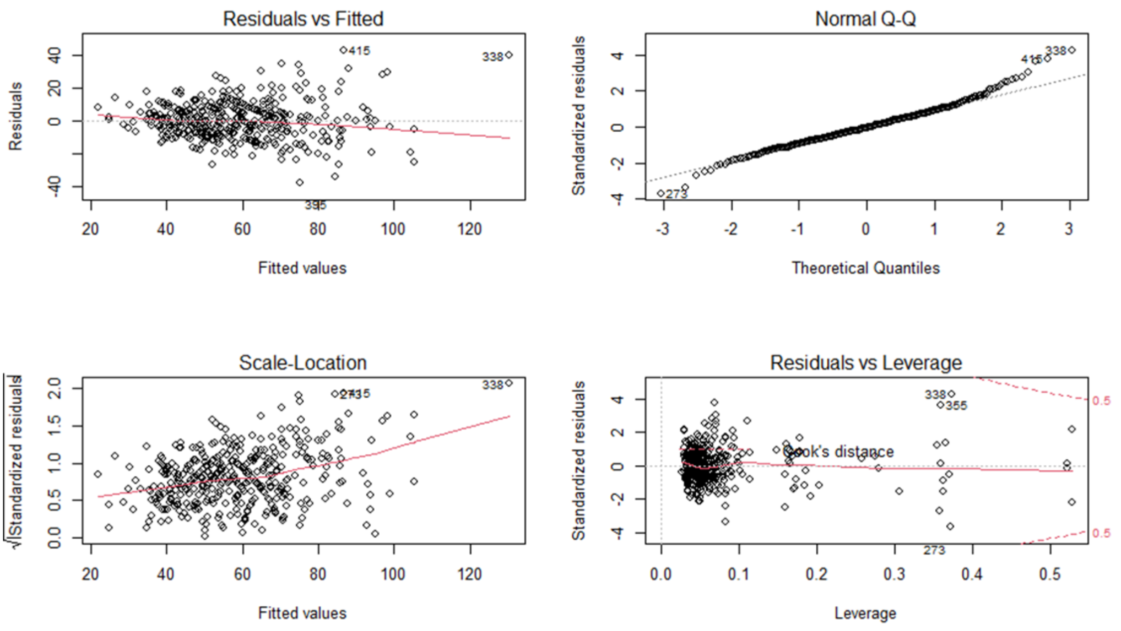

Supplementary figure 2: Goodness of fit analyses of the predicted vs. observed lumpectomy size models.
